# Hot Weather

**Published:** 1874-07

**Authors:** 


### HOT WEATHER.

Now that hot weather is upon us, people will become imprudent as well as impatient, and remove their heavy flannels without supplying themselves with lighter ones. In this manner, are nearly all the colds, influenzas and catarrhs contracted, so prevalent at this season of the year. As yet, we have had but one or two really warm days, upon the advent of which, doubtless, many stripped themselves of their underwear, and went lightly clad in anticipation of a long heated term. Their anticipations have not been realized, but their "colds" have ; for, save the two days mentioned, the weather has been unusually cold and chilly. In this climate, flannels should *always* be worn next the skin—both summer and winter. No one is secure without them.

